# The evolution of moral progress and biomedical moral enhancement

**DOI:** 10.1111/bioe.12592

**Published:** 2019-05-20

**Authors:** Ingmar Persson, Julian Savulescu

**Affiliations:** ^1^ Gothenburg University Gothenburg Sweden; ^2^ Oxford Uehiro Centre for Practical Ethics University of Oxford Oxford United Kingdom

**Keywords:** evoliberal evolution, moral bioenhancement, moral enhancement, moral inclusivism, moral progress

## Abstract

In *The Evolution of Moral Progress* Allen Buchanan and Russell Powell advance an evolutionary explanation of moral progress by morality becoming more ‘inclusivist’. We are prepared to accept this explanation as far as it goes, but argue that it fails to explain how morality can become inclusivist in the fuller sense they intend. In fact, it even rules out inclusivism in their intended sense of moral progress, since they believe that human altruism and prosocial attitudes are essentially parochial. We also respond to their charge that the possibility of moral enhancement by biomedical means that we have defended in numerous publications assumes that moral attitudes are biologically hard‐wired to an extent that implies that they are resilient to the influence of cognitive or cultural factors. Quite the contrary, we think they are more open to such influence than they seem to do.

## 1 INTRODUCTION

In *The Evolution of Moral Progress,* 1Buchanan, A., & Powell, R. (2018). *The evolution of moral progress*. New York: Oxford University Press. Unprefixed page references are to this book. Allen Buchanan and Russell Powell target the apparent problem that ‘evolutionary accounts of morality paint a pessimistic picture, suggesting that certain types of moral progress are unrealistic or inappropriate for beings like us’. According to such accounts, ‘humans are said to be “hard‐wired” for rather limited moral capacities’. However, they argue that the evolution of human morality and our significant social and political achievements instead suggest a ‘dynamic, biocultural theory of moral progress that highlights the interaction between adaptive components of moral psychology and the cultural construction of moral norms and beliefs’.2Buchanan, A., & Powell, R. (2018). The evolution of moral progress. New York: Oxford University Press. Online version doi:10.1093/oso/9780190868413.001.0001, Abstract.


In particular, Buchanan and Powell advance an evolutionary explanation of moral progress in the direction of what they call ‘inclusivist morality’. According to this explanation, human beings have evolved a moral capacity that manifests itself in an inclusivist morality in suitable social, cultural and institutional conditions. It is a combined normative and scientific analysis of the nature and prospects of moral progress, informed by cutting edge evolutionary biology. Not only are we in wholesale agreement with the importance of their target—understanding moral progress—but also with their leading idea that conditions in the social environment can facilitate the development of inclusivist morality. This is a significant and comprehensive work and space precludes our discussing the numerous points of agreement. Instead, we will focus on their discussion of the extent to which moral responses are biologically hard‐wired and the prospects of biomedical moral enhancement (BME). We will focus on points of disagreement. We argue that Buchanan and Powell fail to explain how a morality that is inclusivist in a full sense could develop, and moreover, that they take an overly pessimistic view of the role BME could play in the project of moral progress.

Their key concept of inclusivist morality refers to ‘attitudes and behaviors that extend moral regard or equal basic moral status beyond the narrowest confines of the group’ (64), ‘to all human beings and even to some non‐human animals regardless of their group membership or strategic capacities (i.e., their ability to contribute to or disrupt cooperation)’ (62). The central hypothesis of their evolutionary theory is that an exclusivist moral response is an ‘adaptively plastic’ trait; namely, a ‘conditionally expressed trait that develops only when cues that were in the past reliably correlated with outgroup predation, exploitation, competition for resources, and disease‐transmission are detected’ (189). Typical exclusivist responses like ‘aggression, antipathy and distrust’ are elicited by these cues, and they

reduce the chances of mutually beneficial interactions with neighboring groups, such as trade, mate exchange and alliances, and also increase the chances of dangerous, belligerent, mutually destructive interactions with foreigners. (190)

Thus, moral exclusivity is quite costly populations exhibiting it.

They contrast two conceptions of morality: a ‘strategic conception of morality’, according to which ‘morality [is] simply a matter of self‐interested reciprocal restraints’ (57) and a ‘subject‐centered’ conception, according to which other individuals have moral value or significance not simply as means of furthering the self‐interest of agents, but in themselves. We find their subject‐centred conception quite obscure for several reasons. Buchanan and Powell suggest that, according to this conception, moral status is based on ‘some inherent property of individuals, such as sentience, rationality’ (293). These are of course very different properties. They seem to have sentience in mind when they liken their inclusivist morality to Peter Singer's ‘expanding circle’ of moral concern which extends moral status to all animals endowed with sentience (62). But in the case of human rights the preferred alternative seems to be rationality. In the discussion of this, they affirm:

The best explanation of why certain rights are appropriate for human beings is that, given what humans are like [rational beings] … they need these rights to have a form of life that is of exceptional intrinsic value. (296)

But from the fact that it is appropriate or necessary for someone to have some property, for example, rights, to attain some end, it evidently does not follow that that individual *has* the property. The world is not that ideal. Further problems crop up when they want to include among right‐bearers all disabled humans and humans who might exist in the future, since the former may be incapable of lives ‘of exceptional intrinsic value’, whatever rights they might be accorded, and the latter do not exist and cannot have any rights for that reason.

Such unclarities might seem critical because Buchanan and Powell regard ‘the modern human rights movement’ as ‘arguably the most robust instance of progress in inclusivity’ (379). But it should be admitted that this movement may mark important moral progress even if human rights rest on shaky foundations for it might plausibly be claimed that states preceding it—with blatant racist and sexist features—were even worse: their norms being further from being right, the grounds of the norms being less sound, and the consequences of applying the norms worse. So, let us put these misgivings about the modern human rights movement aside.

## 2 THE EXPLANATION OF MORAL INCLUSIVISM

We agree with Buchanan and Powell that there are social conditions that undermine the exclusivist responses of hostility and distrust, but we want to stress that this is not sufficient for a spread of moral inclusivism. The reason is that we think they are right to hold that ‘there is every reason to think that attitudes toward out‐groups would, prehistorically and historically, have been governed by strategic self‐interest, rather than genuinely subject‐centered considerations’ (190). But if this is so, undermining exclusivist responses is not sufficient for moral inclusivism to extend to the out‐groups. It may be enough for attitudes of *strategic self‐interest* to reach them—that is, it may be enough to establish mutually advantageous exchanges with the outsiders. But moral inclusivism is about extending *the subject‐centred conception* to encompass them; it is about viewing them as having moral significance *in themselves*, not merely as means of furthering the self‐interest of members of the relevant in‐group.

As Buchanan and Powell explain, inclusivist morality represents a type of moral progress to the effect that ‘sympathy is felt not just toward members of one's own family or group but toward suffering beings generally’ (55). But in circumstances in which subject‐centred morality is restricted to the in‐group and attitudes to out‐groups are ‘governed by strategic self‐interest’, the disappearance of exclusivist responses of hostility and distrust to out‐groups, implies only that the road is paved for mutually beneficial exchanges with those outsiders who have something useful to offer to the in‐group. But not being excluded from morality, strategically conceived, does not imply being included within the scope of a subject‐centred morality. Such a non‐exclusion of outsiders is compatible with viewing them as having no intrinsic moral significance, as being moral indifferent unless they can make a positive contribution to the self‐interest of the in‐group.

Certainly, we agree that we humans have what Buchanan and Powell call a ‘capacity for open‐ended normativity’, a ‘capacity to reflect on and revise our moral norms and modify our behavior accordingly’ (180). But in the circumstances under discussion we do not see why this capacity should yield more than an extension of the strategic conception of morality to outsiders who are no longer perceived as threats.

They are aware of this point when they write that it is not the case that ‘moral developmental environments in which out‐group threats are diminished are automatically conducive to deep forms of moral inclusion’ (190). But the problem goes deeper than this concession admits: it is not merely that Buchanan and Powell's ‘evolutionary theory of moral inclusivism’ fails to adequately explain moral inclusivism in the stipulated sense: it even seems to *rule it out*. For they claim that

in‐group favoritism appears to be evolutionarily primitive and hence less culturally and situationally variable than out‐group antagonism; and in‐group biases (in terms of empathy, trust, cooperative tendencies, etc.) result in very significant forms of discrimination against out‐group members even where they do not translate into active out‐group hostility or derogation. Thus, even if moral developmental environments are conducive to prosocial interactions between groups, this does not mean that these interactions will necessarily be guided by robustly inclusivist moral commitments of the sort that characterize recent expansions of the moral community. (190–191)

‘Altruism and exclusivism appear’, they maintain, ‘to be two sides of the same adaptive coin’ because ‘morality was forged in the crucible of intergroup conflict’ (209). This link makes them sceptical of our proposal to enhance altruism by biomedical means:

Enhancing the biological basis of altruism may thus amount to sharpening both sides of a double‐edged sword: by strengthening the biological (hormonal, genetic, etc.) basis of altruism, we may unavoidably exacerbate antisocial attitudes and behaviors toward out‐groups. (364)

This overlooks the possibility that, though conflict was necessary for the *development* of altruism, it is not necessary for its *persistence*. Consider adaptively plastic traits such as exclusivist responses. Buchanan and Powell claim that organisms will develop such exclusivist dispositions only if they face threats in their environment. But the fact these exclusivist dispositions would not have been acquired if the environment had not been such that their actualization had been useful does not mean that they cannot persist when there is no need for them to be actualized any longer because the environment has become friendly and peaceful. They are likely to persist for a long time, ready to be actualized if out‐group threats should again appear on the horizon.

This is a respect in which their simple model of an adaptively plastic trait—the protective armour of some species of water fleas—is misleading. The water fleas develop this armour ‘only when they find themselves confronted with the high probability of a predator‐rich environment’ (189). But once it has developed, they carry their armour around even if the environment becomes safer. A more suitable model for exclusivist responses would be a disposition or capacity to exhibit some form of protection that is actualized when a predator is confronted, but otherwise remains dormant. In the case of the water flea armour, there is no counterpart to this stage of having a disposition that has been actualized in the past, but is not actualized at the present time. Such a counterpart is, however, necessary to do justice to exclusivist responses.

It is plausible to hold that altruism within groups would not have evolved unless these groups were involved in conflicts with other groups. But that does not mean altruism must die out if the groups in which it has become dominant are victorious, and they encounter few or no threats from out‐groups. In these circumstances, exclusivist responses tend to be replaced by strategic cooperation with out‐groups, and altruism is likely to spread beyond the in‐group to these cooperative out‐groups due to their beneficial input. Since this spread of altruism is conducive to the stabilization of cooperative activities that are beneficial to all parties, it is not likely to diminish. Such a spread of altruism is necessary for a genuine moral inclusivity in which moral status and concern are extended beyond the in‐group.

Thus, even if ‘parochialism was a necessary condition for the evolvability of human altruism’ (363), it does not follow that altruism is by necessity ‘tightly bound to partiality’ (362) in all circumstances. We have elsewhere argued against Paul Bloom and Jesse Prinz that empathy, conceived of as imagining what someone else feels, is under voluntary control.3Persson. I., & Savulescu, J. (2018). The moral importance of reflective empathy. *Neuroethics, 11*(2), 183–193. By contrast, Buchanan and Powell explicitly side with Prinz (362). If we voluntarily imagine vividly what is like for someone else to feel pain, for instance, this is apt to evoke in many of us sympathy and an intrinsic desire that this pain be relieved, which is what we take altruism to consist in. But there are various factors that are likely to prevent us from undertaking such acts of imagination—if we take others to be hostile, physically disgusting, members of some despicable ethnic group, and so on. It is therefore essential that such blockers be undermined by social change or reflection.

This brings out that we regard cognitive factors as having an important role to play in extending the range of our sympathy or altruistic concern for other subjects. Buchanan and Powell note (361–362) that we acknowledge that the positive effects of putative biomedical moral enhancers like oxytocin on such prosocial attitudes tend to be restricted to in‐groups.4Persson. I., & Savulescu, J. (2012). *Unfit for the future: The need for moral enhancement*. Oxford: Oxford University Press; pp. 119–120. But they omit mentioning that we contend that this restrictive tendency can be counteracted by moral reasoning and, thus, that BME ‘would have to go hand in hand with reasoning which undercuts race, sex etc. as grounds for moral differentiation’.5Ibid. See also our reply to an earlier article by them: Persson. I., & Savulescu, J. (2017). Moral hard‐wiring and moral enhancement. *Bioethics*,* 31*(4), 286–295; p. 292. Buchanan and Powell thank us for comments on their work, but curiously do not refer to this article, which is a detailed critique of their work. So, we are in agreement with their claim: ‘Flexibility and cultural sensitivity is built, as it were, into the adaptive design of human morality’ (356).

They claim that a position like ours implies that human beings are now ‘bumping into the limits’ (188) of their capacity for moral inclusion, but it can now be seen that our position allows for moral inclusion in the form of altruism to expand further than their position does. We believe that BME in conjunction with imagination guided by sound moral reasoning about what properties are and are not morally relevant can do more to overcome the parochialism of our spontaneous altruism than they seem to permit, though it could not reasonably make it as strong toward strangers as it is toward those who are near and dear to us.

Even if BME is not by itself sufficient to enhance the altruism of most of us to the degree required to deal with the moral mega‐problems of our time like anthropogenic climate change and the stupendous global inequality, we think that it might still be necessary, unless we are by nature exceptionally altruistic and have been prepared to undergo extensive cognitive training, like Matthieu Ricard, who for many decades has enhanced his capacity for compassion by Buddhist meditation.6See Ricard, M. (2015). Chapter 21 and 22. In M. Ricard, *Altruism. The science and psychology of kindness* (pp. 247–270). London: Atlantic Books. It certainly seems that some people—in particular psychopaths—are incapable of exhibiting noticeable altruistic concern even when confronted by the most vivid representations of the suffering of others. For these people, and to a less extent for more normal people, BME might be the only feasible means of boosting their altruism to the requisite degree.7Persson & Savulescu (2017), Op. cit. note 4, pp. 291–292.


In this context, it should be observed that Buchanan and Powell's adaptively plastic exclusivist responses are also likely to be within the purview of BME as they are biologically hard‐wired. In principle, it should be possible to weaken these responses by BME, so that it takes more of hostile or threatening behaviour from others to call forth exclusivist responses. This would be a supplementary way of fighting exclusivism, in addition to ameliorating the social conditions that provoke such behaviour.

To summarize this part of our reply: We do not object to the part of their account that construes exclusivist responses as adaptively plastic traits, though we think it is insufficient to explain moral inclusivism, and we want to insist that it does not rule out that BME has a significant role to play. However, we do find the claim about the inescapable parochiality of altruism that they tack onto this account implausible and incompatible with moral inclusivism. This should make it clear how mistaken they are when they characterize us as ‘evoliberals’ who subscribe to the view that ‘human beings are hard‐wired by evolution for exclusivist moralities’ (381). We can accept their account of the plasticity of exclusivist responses, and we do not think that the parochialism of spontaneous altruism is hard‐wired and resilient to cognitive factors to the extent they do.

## 3 THE EXTENT OF MORAL PROGRESS AND THE ROLE OF BME

Buchanan and Powell make the conciliatory remark that they ‘see no in‐principle objection to using biomedical technologies in conjunction with cultural modes of moral enhancement to bring moral motivations and behaviors in line with the norms we have come to endorse’. But they immediately add that their analysis leads them ‘to the pessimistic conclusion that BME is unlikely to play a necessary or even major role in the future of moral progress or in solving the greatest moral dilemmas of the coming centuries’ (373). Our view is that it is too early in the day to condemn BME as ineffectual: too little research has been made into the matter for either a favourable or unfavourable judgment on its potential effectiveness. We argue that the contemporary moral problems are so grave that it is worth looking into all possible means of moral improvement, including BME. In our publications, we have emphasized moral enhancement by biomedical means, not because we believe that it will definitely be more effective than moral enhancement by more traditional means, but because it has been a neglected or rejected alternative.

Buchanan and Powell are optimistic about the moral progress that has been made so far as well as about the moral progress that we could expect in the future:

…[T]he last few centuries have witnessed a dramatic shift to more subject‐centered theories of morality. […] It is not much of a stretch, therefore, to think that the moral circle could expand yet further, under the right social and epistemic conditions, to include anonymous individuals who will come to exist long after all existing people are gone. (360)

They think this expansion of the moral circle could be achieved ‘by institutional innovations that create incentives for cooperation, which … are conducive to the development of less selfish moral norms and attitudes’ (361). We do not see what the ‘institutional innovations’ could be that could make people morally more concerned about individuals distant in time and space. (With respect to future people, cooperation is even *in principle* impossible.) They claim that ‘the threat of punishment exerts a much stronger influence on prosocial behavior than does oxytocin or other BME variables’ (371). But the threat of punishment is not anything that increases our altruistic *motivation*; it makes use of our selfish motives to change our behaviour in ways that are less harmful to others, to avoid the costs of punishment to ourselves. Certainly, if people in affluent countries were punishable by law if they omitted various contributions to mitigating climate change or to alleviating the plight of the global poor, their contributions to these causes would be likely to grow. However, the hitch with this proposal is, as we point out (2012: Chapter 7, 2017, pp. 293–294), that as long as affluent people are as little altruistically concerned as they are at present about the global poor and people in the more remote future, the requisite laws are unlikely to be approved and passed.

Buchanan and Powell accuse us of making ‘much of the fact that there has been little moral progress in over 2500 years since the first great teachers of morality’, whereas they think that there has ‘been monumental moral progress over the last few centuries’ (372). But we do not claim that there has been ‘little’ moral progress in this period; we make the *comparative* claim that this progress ‘is nowhere near matching the degree of technological progress during the same period’ (2012, p. 106). This comparative claim is supported, for instance, by such facts as that, while we can still learn important things about morality from studying the teachings of Buddha and Plato's Socrates, what their contemporaries thought about physics and chemistry, say, is hopelessly obsolete. Presumably, this is because human nature has not changed much in 2,500 years. Certainly, many states and societies have become more civilized, but this is not to be confused with their citizens having undergone fundamental moral improvement, by having become more benevolent, fair‐minded, less selfish, deceitful, and so on. Surely, we are not to believe that people underwent a fundamental moral change in the span of the century it took for the wild west of the USA to transform into civilized society. That civilization of a society does not imply fundamental moral improvement is indicated by the fact that seemingly civilized societies as late as in the 20^th^ century regressed to the most horrendous crime of genocide which has stained all human history (2012, p.105). Does not such regresses make it hard to believe in a ‘monumental moral progress’?

Although it is not incontestable, our comparative claim about progress is easier to support, and also more relevant for our argument about the current mismatch between our moral and technological capacities. We were arguing that because our rapidly expanding technological capacities have left our more constant natural capacities for moral concern far behind, the latter may need a boost by biomedical means. It would be grossly implausible to claim that there has ‘been monumental moral progress over the last few centuries’ as regards these capacities for moral concern when, for instance, the majority of people in affluent countries are complacent about the fact that more than half of the Earth's human population is indecently poor.

This should make it clear that our disagreement with Buchanan and Powell about the amount of moral progress is to a considerable extent due to the fact that we are talking about different things. Their claim about the ‘dramatic shift to a more subject‐centered theories of morality’ that has ‘culminated in the robust system of international human rights’ (360) is an assertion about an *ideological* change which has resulted in certain *official declarations*. We are talking about the *motivation* of people and their actual *behaviour*. Now we are not saying that there is a ‘massive’ rift between these phenomena, as Buchanan and Powell put it (368), but we do think there is an *important* rift between them.8Ibid. p. 289. The supposedly ‘robust system of international human rights’ is regularly grossly violated by some countries, for instance, recently by Myanmar. And even in nations in which human rights and the egalitarian ideology have a more secure foothold, like Western Europe and the USA, there are evidently strong racist attitudes underneath the surface. The latter is something that Buchanan and Powell themselves appear to concede when they say about the situation in the USA that ‘implicit forms of prejudice and stereotype remain pervasive and explicitly racist attitudes are still prevalent in certain subpopulations’ (155).) Consequently, human motivation and behaviour in general are clearly not abreast with the lofty moral aspirations or ideals expressed by some laws and political declarations.

We concur with them that it is dubious whether BME ‘interventions could be implemented with sufficient rapidity on a sufficiently massive scale […] to address imminent catastrophic threats’ (364). However, our hypothesis is rather that such an implementation may well be *necessary* to neutralize these threats. This does not imply that such an implementation is *likely* to occur; perhaps what is most likely is that we shall walk into some disaster.9Ibid. p. 294. Buchanan and Powell believe that we

are committed to an inconsistent view about the power of culture: on the one hand, they think cultural innovations are too feeble to solve major problems humanity now faces; on the other, major cultural innovations—including unified political will and agreement on the propriety of biomedical interventions for moral improvement—would be necessary if the biomedical interventions they propose were to be achieved on a sufficient scale to make a difference. (381)

We would indeed have an inconsistent view if we had thought (a) that human beings had *no* motivation whatsoever to be moral, and yet (b) could be motivated to arrange cultural innovations designed to improve them morally. But we do not think that humans lack *all* moral motivation, only that it is not strong enough for them to conduct themselves in a manner necessary to deal with their moral mega‐problems, unless it is enhanced by certain means. This is compatible with their motivation being strong enough for them to make certain preparations in advance to strengthen their motivation. Human beings are often in situations in which they can predict what choices they will face in the future. They may then realize that, unless they take certain steps, they will backslide in the heat of action and fail to do what they now, in a cool hour, are aware that they ought to do. For instance, in a cool hour they may realize that they ought to cut down on their driving and flying in order to mitigate climate change, but they may also predict that when they enter situations in which it is convenient for them to drive or fly they are likely to backslide, unless their moral motivation is enhanced between now and then. In such situations their moral motivation to apply suitable BME, were it available, to themselves could be strong enough to do so, though it is not strong enough to withstand backsliding at the time of action.10For further elaboration of this argument, see Persson. I., & Savulescu, J. (2019). Moral bioenhancement—not a lever without a fulcrum. *Neuroethics, 12*(1), 19–22.


Moreover, every human society must have a set of moral norms to survive. What norms we choose to implement and use to educate and socialize children can determine the part BME has to play in moral education. One of the great challenges facing post‐religious society is to develop a secular set of moral norms to serve as a basis for education and social organization. We have suggested co‐operation, tolerance, non‐discrimination and justice are important parts of a common secular morality. BME may have a role to play to augment moral education, just as cognitive enhancers may have a role to augment conventional education, as is the case with the use of methylphenidate (Ritalin) in attention deficit disorder, a condition that affects up to 10% of children. Ritalin not only improves self‐control and ability to delay gratification, but it also reduces impulsive violence, a form of BME.

Consequently, our position is not inconsistent, but as we concede,11Op. cit. note 3, p. 124. there *is* a boot‐strapping problem that we do not see how to solve satisfactorily. It consists in the fact that it is morally imperfect human beings who will have to decide to do research in order to discover effective biomedical means of moral enhancement and to apply them properly to themselves. There is no guarantee that they will succeed in either respect, but if they succeed in taking the first steps to enhancing themselves morally, the probability that they will succeed in taking further steps increases. Notice, however, boot‐strapping problems face all means of moral enhancement that humans have to subject themselves to, not just biomedical ones.

So urgent is the need for moral progress that we should explore all avenues in a complementary manner: social, legal, institutional and cultural, but also psychological and biological. Buchanan and Powell stress the social and institutional. They are pessimistic of the possibility of BME. We do not see such enhancement as a panacea, but it may be an important part of moral progress. Only further research— ethical and scientific—will enable us to tell.

## CONFLICT OF INTEREST

The authors declare no conflict of interest.

